# Texas State Medical Association

**Published:** 1898-04

**Authors:** Bacon Saunders

**Affiliations:** President; Fort Worth, Texas


### Texas State Medical Association.

FORT WORTH, TEXAS, March 1, 1898.

*To the Members of the Texas State Medical Association and the Profession of the State at Large:*

The next annual meeting of the State Medical Association will convene in the city of Houston on Tuesday, April 26, 1898. The reasons why every doctor in the State who is now a member or has been a member or contemplates becoming a member of the State organization, should attend this meeting are so cogent that all must appreciate them.

Questions of the most vital import, not only to every physician, but to every citizen of the State as well, will come before that convention for consideration and solution, if possible.

Houston is accessible to all; a delightful place to meet at that season, and its profession is anxious to extend to us the hospitality of their city. The local chairman informs me that he will probably secure a rate of one fare for the round trip.

If every doctor in Texas, who loves his State and regards her present greatness and future possibilities and is impressed with the weight of responsibility resting on our profession in shaping its future destiny will come to that meeting, it will be one the like of which will not be soon forgotten.

Further information will be found in the circular of the Chairman of the Committee of Arrangements, Dr. Jos. A. Mullen, Houston, Texas, and the official programme of Dr. H. A. West, Secretary.

BACON SAUNDERS, President.

